# Analysis of the Impact of Selected Vitamins Deficiencies on the Risk of Disability in Older People

**DOI:** 10.3390/nu13093163

**Published:** 2021-09-10

**Authors:** Wassim Gana, Arnaud De Luca, Camille Debacq, Fanny Poitau, Pierre Poupin, Amal Aidoud, Bertrand Fougère

**Affiliations:** 1 Division of Geriatric Medicine, Regional University Hospital Centre, 37000 Tours, France; f.poitau@chu-tours.fr (F.P.); a.aidoud@chu-tours.fr (A.A.); bertrand.fougere@univ-tours.fr (B.F.); 2 Geriatrics Mobile Units, Regional University Hospital Centre, 37000 Tours, France; c.debacq@chu-tours.fr (C.D.); p.poupin@chu-tours.fr (P.P.); 3 Nutrition Mobile Unit, Regional University Hospital Centre, 37000 Tours, France; a.deluca@chu-tours.fr; 4 Inserm UMR 1069, Nutrition, Croissance et Cancer, 37032 Tours, France; 5 Education, Ethics, Health (EA 7505), Tours University, 37000 Tours, France

**Keywords:** malnutrition, micronutrients, vitamins, disability, frailty, sarcopenia, older, rehabilitation

## Abstract

Vitamin deficiencies have a serious impact on healthy aging in older people. Many age-related disorders have a direct or indirect impact on nutrition, both in terms of nutrient assimilation and food access, which may result in vitamin deficiencies and may lead to or worsen disabilities. Frailty is characterized by reduced functional abilities, with a key role of malnutrition in its pathogenesis. Aging is associated with various changes in body composition that lead to sarcopenia. Frailty, aging, and sarcopenia all favor malnutrition, and poor nutritional status is a major cause of geriatric morbidity and mortality. In the present narrative review, we focused on vitamins with a significant risk of deficiency in high-income countries: D, C, and B (B6/B9/B12). We also focused on vitamin E as the main lipophilic antioxidant, synergistic to vitamin C. We first discuss the role and needs of these vitamins, the prevalence of deficiencies, and their causes and consequences. We then look at how these vitamins are involved in the biological pathways associated with sarcopenia and frailty. Lastly, we discuss the critical early diagnosis and management of these deficiencies and summarize potential ways of screening malnutrition. A focused nutritional approach might improve the diagnosis of nutritional deficiencies and the initiation of appropriate clinical interventions for reducing the risk of frailty. Further comprehensive research programs on nutritional interventions are needed, with a view to lowering deficiencies in older people and thus decreasing the risk of frailty and sarcopenia.

## 1. Introduction

Malnutrition includes undernutrition and micronutrient deficiencies. There is a lack of consensus regarding the definition of malnutrition: the European Society of Clinical Nutrition and Metabolism’s guidelines define it as “a state resulting from lack of intake or uptake of nutrition that leads to altered body composition and body cell mass leading to diminished physical and mental function and impaired clinical outcome of disease” [1]. According to the recent Global Leadership Initiative on Malnutrition consensus statement, malnutrition can be diagnosed when at least one phenotypic criterion (non-volitional weight-loss, low body mass index (BMI), or reduced muscle mass) and one etiologic criterion (reduced food intake/malabsorption or severe disease with inflammation) are met. Prior to the diagnosis of malnutrition, the criteria for a nutritional risk (according to a validated nutritional risk screening tool) must be met. Older people are at risk of malnutrition if their oral food intake is markedly reduced (e.g., below 50% of the requirement for more than three days) or if they have risk factors (acute disease, neuropsychological problems, immobility, chewing problems, swallowing problems) that either reduce the dietary intake or increase the dietary requirement [2]. In a cross-sectional study of generally healthy older adults, 45% of the participants had inadequate intake and micronutrient deficiencies—resulting in a weight loss of 100 g for every 1% decrease in total calorie intake [3]. Approximately 5–10% of community-dwelling older adults, 50% of older adults in rehabilitation facilities, 20% in residential care, and 40% in hospitals are undernourished [4]. Malnutrition has many consequences. Malnourished older inpatients have an increased risk of mortality, a longer length of hospital stay, higher hospital readmission rates, and higher incidences of infection and impaired wound healing. In community-dwelling older adults, the nutritional risk may lead to more consultations with the family physician, more hospital admissions, and an increased risk of falls [5]. Malnutrition is associated with poor quality of life [6] and reduced functional abilities and limitations in basic activities of daily living (ADL) [7,8].

Malnutrition, therefore, represents a major burden for geriatric populations and is becoming a priority for the promotion of healthy, successful aging. Prevention of the disability related to dependence in ADL [9] is a constant concern in the healthcare of older people. Given the heterogeneity of the aging process, the prevention and treatment of dependency in older adults are nevertheless challenging. Disability is a complex, dynamic phenomenon characterized by frequent transitions between independent and dependent states [10,11]. Frailty is a preclinical or clinically silent stage of disability, characterized by reduced functional abilities and a greater likelihood of becoming dependent in stressful situations [12,13]. This condition is common among older adults; the estimated prevalence is around 15% for adults aged 65 and older, but it increases to 18% to 40% with age or in other contexts (e.g., hospital settings) [14].

Malnutrition has a key role in the pathogenesis of frailty. Boulos et al. [15] reported that malnutrition and the risk of malnutrition were associated with an almost four-fold increase in the risk of frailty. In another study, 90% of patients at risk of malnutrition were either pre-frail or frail [16]. The French Society of Nutrition and the French National Authority for Health went further by considering that all malnourished elderly patients are frail [17]. Malnutrition and frailty have common sociodemographic, physical, and cognitive risk factors [15]. Nutritional risk also increases the consequences of frailty, including the risk of hospitalization and loss of independence.

In this pre-morbid state, aging is associated with various changes in body composition (including weight changes, loss of muscle mass, and increase in fat mass), which may lead to under-diagnosis of malnutrition [18,19]. These changes result in sarcopenia or a loss of function and significantly worsen the quality of life. Sarcopenia is correlated with increased functional disability in ADL, a greater risk of falls, frailty syndrome, and dependence [20]. Sarcopenia is highly prevalent in rehabilitation institutions or acute geriatric units [21]. It frequently co-exists with malnutrition in older patients: the decline in food intake contributes to weight loss, muscle mass loss, and a decline in strength and physical function [22]. Sarcopenia impairs metabolic adaptations to stress and disease. Hence, there is a significant degree of overlap between sarcopenia and the frailty promoted by inflammaging [23].

Even though aging is a physiological process, it is accompanied by stimulation of the inflammatory system; this so-called “inflammaging” is particularly associated with greater production of interleukin (IL)-6 [24]. This molecule increases catabolism and promotes the development of insulin resistance. The biological association between sarcopenia and frailty manifests itself through abnormal activation of the inflammatory system, increased cytokine release [25], neuroendocrine dysregulation (with elevated serum cortisol levels and low levels of the anabolic insulin growth factor, growth hormone, and sex hormones), reduced physical activity, and reduced food intake. All these mechanisms are involved in sarcopenia, the loss of muscle function, and therefore frailty [23].

The nutritional health of older people is a key issue. Aging, frailty, and sarcopenia all favor malnutrition. Conversely, poor nutritional status is a major cause of geriatric morbidity and mortality [26]. Factors such as acute illness or hospitalization can disturb this precarious balance. Reduced food intake and impaired compensatory mechanisms can make it more difficult for older people to meet their nutrient needs—particularly with regard to micronutrients [2,27,28].

As soon as the glycogen reserves are depleted (i.e., after a few hours of stress or fasting), the body draws on its own proteins (i.e., muscle) to meet energy needs via gluconeogenesis. Hence, the body enters a catabolic state. These energy reserves are recruited through the action of pro-inflammatory, anorexigenic cytokines (e.g., tumor necrosis factor alpha, IL-1, and IL-6) [29,30], which decrease albumin synthesis and induce protein breakdown and lipolysis [29]. These phenomena partly explain the frequent state of malnutrition observed in inpatients.

The nutrients that have been more consistently linked to sarcopenia and frailty are proteins and vitamins D, B9, C, and E [31,32]. Other vitamins (such as the water-soluble B vitamins) are also involved in cellular aging processes involved in the onset of frailty (e.g., inflammation, oxidative stress, and immune pathways) [33].

In the present narrative review, we focused only on vitamins D, C, E, and B (B6/B9/B12) in older adults because these are particularly at risk of deficiency in high-income countries. Regarding vitamin E, it is the main lipophilic antioxidant agent, with a metabolism synergistic to vitamin C. We first look at how these vitamins are involved in the biological pathways associated with sarcopenia and frailty. We then discuss the importance of the early diagnosis and management of these deficiencies. Lastly, we summarize possible ways of detecting malnutrition early, with a view to reducing impairment and disability.

## 2. Vitamin D

Vitamin D is a pleiotropic, fat-soluble vitamin that is mainly synthesized via exposure to sunlight. Its biologically active form (1,25(OH)_2_D) has a major role in bone metabolism. More recently, research has focused on Vitamin D’s non-skeletal beneficial effects, such as greater muscle strength, low inflammation, immune stimulation, and better mood. Vitamin D insufficiency and deficiency are currently defined as a 25-hydroxy vitamin D (25-OHD) level between 20 and 29 ng/mL and below 20 ng/mL (50 nmol/L), respectively, and these conditions are common in older populations [34]. Patients with fat malabsorption or maldigestion are particularly exposed to vitamin D deficiency. The storage of vitamin D in fat makes it harder to assess the status of this vitamin, especially in people with a high fat mass. Indeed, most older people have a higher body fat mass due to physiological changes in body composition caused by the aging process. This modification of body composition leads to a decrease in the bioavailability of vitamin D due to its storage (sequestration) in the adipose tissue [35]. Furthermore, older adults have a higher prevalence of low D vitamin levels due to low dietary intake and reduced ultraviolet irradiation of the skin. The main food sources of vitamin D are fatty fish, oily fish, fish liver, red meat, liver, egg yolks, cheese, milk, and mushrooms [36,37]. The recommended dietary allowance (RDA) ranges from 600 IU/day (for people aged 1 to 70) to 800 IU/day (for people aged 71 and over). These RDAs correspond to a serum 25-hydroxyvitamin D level of at least 20 ng/mL (50 nmol/L) [38] (Table 1). 

The results of relevant epidemiological studies have suggested that vitamin D deficiency in older adults is independently related to low muscle mass and function, poor physical performance, and the risk of falls [88,89]. Visser et al. showed that deficient individuals (serum [25-OHD] > 25 nmol/L) had lower grip strength 3 years later [90]. Similarly, vitamin D deficiency has been correlated with a decrease in walking time or time to complete a repeated chair stand [88]. Thus, vitamin D deficiency exposes a person to sarcopenia. Other studies have focused on the link between vitamin D deficiency and the risk of other geriatric comorbidities, such as frailty. Low vitamin D status has been associated with an increased risk of becoming frail [91] and with the severity of frailty [92]; these outcomes appear to be promoted by the development of sarcopenia. Various mechanistic links have been identified. Vitamin D regulates skeletal muscle function directly by affecting the muscle’s anabolic state and energy metabolism [93]. Vitamin D also contributes to reducing the low-grade inflammation associated with sarcopenic older adults [94] (Table 2). Vitamin D is also involved in the regulation of oxidative stress as an antioxidant (mitochondrial activities, stress-related protein oxidation, lipid peroxidation, and DNA damage) [95]. Sepidarkish et al. [96] reported in a meta-analysis that vitamin D supplementation can improve oxidative stress parameters. In another study, authors showed that high-dose administration of vitamin D3 to deficient older women increased total antioxidant capacity (TAC) levels [97]. The results are controversial; however, the literature is scarce on this topic [96].

Among older patients in rehabilitation centers, the prevalence of vitamin D deficiency is around 90% [46,47]. Kiebzak et al. [47] evaluated the relationship between vitamin D status and short-term rehabilitation progress in hospitalized patients. Almost all patients had a suboptimal serum 25-OHD concentration (94%), and 11% were deficient. Higher serum 25-OHD concentrations were a predictor of better functional recovery after rehabilitation. Among community-dwelling older adults, the prevalence of vitamin D deficiency is between 17% and 56% [41]. This prevalence increases to 80–90% in people in nursing homes [48,49].

Although the consequences of vitamin D deficiency on skeletal muscle function have been extensively studied, the effects of supplementation remain subject to debate. Several randomized clinical trials have been conducted to determine whether oral vitamin D supplementation prevents or reduces poor functional outcomes in older patients. However, the involvement of vitamin D supplementation in rehabilitation outcomes is still not clear. In a recent clinical trial, oral vitamin D3 supplementation did not improve rehabilitation outcomes after acute stroke [104]. However, the initial vitamin D status (which may influence recovery) was not evaluated. The results are also controversial about supplementation on both musculoskeletal health and muscle function. National Institutes of Health (NIH) [36] mentioned that recent systematic reviews concluded that “vitamin D and calcium supplements had no beneficial effects on fractures, falls, or bone mineral density” [171,172]. Another systematic review and meta-analysis of 11 randomized, controlled trials of vitamin D supplementation alone found that the supplements “provided no protection from fractures in older adults” [173]. After our own reviewing of the literature, we suggest that vitamin D supplementation might be beneficial only in patients with a deficiency. However, the data are incongruent, and further evidence of clinical benefit is required [34,105,106]. Furthermore, the NIH concluded about vitamin D supplements and bone health that “all adults should consume recommended amounts of vitamin D and calcium from foods and supplements if needed. Older women and men should consult their healthcare providers about their needs for both nutrients as part of an overall plan to maintain bone health and to prevent or treat osteoporosis” [36].

Vitamin D deficiency in older people is associated with a loss of muscle mass and strength, although supplementation (with or without an associated exercise program) does not appear to increase strength and muscle mass gains [174,175]. However, a recent systematic review found that supplementation with a combination of leucine and vitamin D was associated with a greater lean body mass [176]. At the biological level, 1,25(OH)2 vitamin D3 potentiates the stimulating effect of leucine and insulin, leading to further activation of protein synthesis in murine C2C12 skeletal myotubes [177]. Similarly, the ability of leucine to stimulate muscle protein synthesis was significantly amplified after the co-administration of antioxidants (a mixture containing rutin, vitamin E, vitamin A, zinc, and selenium). These results suggest that vitamins potentiate the positive effect of anabolic factors.

## 3. Vitamin C

Vitamin C (ascorbic acid) is an essential micronutrient for nutritional health. In humans, vitamin C is not synthesized by the body and so must come exclusively from the diet. The main food sources of vitamin C are fruits, vegetables, and potatoes [50]. According to the Institute of Medicine (2000) [53], the RDA ranges from 75 mg/day for adult women to 90 mg/day for adult men. However, increasing the ascorbic acid intake to 100–200 mg/day will maintain normal plasma concentrations [178]. As a water-soluble vitamin, vitamin C is absorbed by active and passive mechanisms in the distal ileum [107]. Vitamin C levels are also dependent on renal excretion and reabsorption [179]. Vitamin C is not stored in the body, and so plasma concentrations are directly correlated with dietary intake. Vitamin C deficiency is often the consequence of low intake or inflammatory conditions. However, there is a difference in vitamin concentration depending on sex. Women have higher plasma concentrations than men. This difference could reflect a larger volume of distribution of vitamin C in women with a higher absolute fat-free mass than men [180]. Vitamin C insufficiency and deficiency are currently defined as a serum level between 11 and 28 µmol/L and below 11 to 11.4 µmol/L, respectively [51,52].

It has long been known that vitamin C deficiency leads to scurvy, with purpura and hemorrhage. However, vitamin C has pleiotropic effects and is involved in physiological processes and the pathogenesis of various other communicable and non-communicable diseases [56]. Epidemiological studies have revealed an association between vitamin C status and overall mortality [119]. The association between cancer/cardiovascular disease mortality and vitamin C was stronger than for any other micronutrient [120,121]. Other studies concluded that people with lower vitamin C plasma levels were at increased risk of cardiovascular disease; however, studies of vitamin C supplementation showed no reduction or only a moderate reduction in the risk of heart disease [122]. Rowe and Carr (2020) [56] reported that the data from high-income countries are quite old and that few studies have examined older populations specifically. In the UK, Bates et al. [58] found that the prevalence of vitamin C deficiency was 14% in people aged over 65 between 1994 and 1995. A study performed in Glasgow in 1992 [57] found a prevalence of 20% in people aged between 25 and 74. In France [59], the prevalence of vitamin C deficiency was 9% in people over 60 between 1995 and 1997. More recently, an American study [51] performed between 2003 and 2004 found a value of 8.4% in people over 20. The prevalence of vitamin C deficiency in geriatric populations (and particularly among inpatients) is uncertain [56]. Further studies on the role of vitamin C in the aging process, age-related diseases, and major geriatric syndromes are therefore required, together with updated epidemiological data specifically on older populations (Table 1).

Vitamin C is an important antioxidant involved in genetic and epigenetic regulation mechanisms [108], oxidative stress, and inflammation. It is very clear that reactive oxygen species (ROS) have a role in aging and age-related diseases [181]. Oxidative stress is associated with sarcopenia [182]. Researchers have observed a positive association between circulating vitamin C levels and measures of skeletal muscle mass in older-aged patients [112]. Similarly, frailty was found to be associated with lower concentrations of antioxidants (e.g., vitamins C and E) [110]. These findings suggest that the balance between antioxidant processes and ROS toxicity is altered during aging. Therefore, vitamin C’s major antioxidant function makes this compound an important topic of research with regard to the loss of autonomy and age-related disability. However, the effectiveness of vitamin C supplementation in sarcopenia or frailty is unclear. Balboa-Castillo et al. [111] concluded that a lower vitamin C intake was associated with a higher risk of frailty in older people, whereas others [123] did not find a relationship between vitamin C supplementation and improved grip strength or the prevention of low-grip strength over a 5-year period. All these conflicting results mean that further studies are required. Vitamin C is also involved in the central nervous system’s normal functions [183], and brain levels of this vitamin are high [56,184]. Some studies have shown that people with a high vitamin C status had lower levels of mild cognitive impairment [114,115,116]. In a systematic review, Travica et al. [117] came to the same conclusion: vitamin C concentrations were higher in cognitively intact participants than in cognitively impaired ones, even though there was no correlation between the vitamin C concentration and the Mini-Mental State Examination score in the cognitively impaired participants. In another study, the same researchers found a significant association between vitamin C plasma concentrations and cognitive function, regardless of whether the source of vitamin C was the normal diet or a supplement [118]. The researchers found an effect of sex on the vitamin C distribution in the plasma and the brain. This sex difference may contribute to sex-associated cognitive ability [185]. These intriguing results require further investigation, and potential confounding variables must be identified (Table 2).

Vitamin C deficiency has important consequences in older adults but appears to be underdiagnosed—especially in frail, hospitalized older adults who often have several disabilities (dementia, loss of autonomy, depression, etc.) and often need help with eating. In this context, a French study [113] concluded that scurvy is not uncommon in hospitalized older adults and that the latter should be given vitamin-C-rich food. There is a need for studies of the prevalence and functional consequences of vitamin C deficiency and tools for preventing and effectively managing this deficiency in older adults.

## 4. Vitamin E

The diet is the only source of vitamin E (α-tocopherol and γ-tocopherol). After absorption in the intestine, this fat-soluble vitamin is delivered to the liver. The hepatic alpha-tocopherol transfer protein recognizes α-tocopherol in particular and transfers it to the plasma [186]. Mulligan et al. [60] found that “the adequate intakes of α-tocopherol for adults are 13 mg/day for men and 11 mg/day for women” [63]. According to the Institute of Medicine (2000) [53], the RDA is 15 mg (35 µmol)/day for adults. Although the correlation between the dietary α-tocopherol intake and the serum α-tocopherol concentration is weak, the European Food Safety Authority [63] considered that a serum α-tocopherol concentration below 12 μmol/L can indicate a deficiency. Vitamin E is mainly found in vegetable oils, nuts, seeds, wheat germ, and some vegetables (avocado and spinach) [60,61,62]. Among older people, the causes of vitamin E deficiency are cholestatic liver diseases, severe malnutrition, fat malabsorption, fat metabolism disorders, cystic fibrosis, mutations in the tocopherol transfer protein, and isolated vitamin E deficiency syndrome [63,64]. The prevalence of vitamin E deficiency is low in high-income countries (0.1% in the USA) [64], while it is sometimes very high in developing countries (up to 55%) [65] (Table 1).

Some models of frailty feature accelerated aging. Indeed, this age-related syndrome is a multimodal, dynamic process. At the cellular level, oxidative stress and impaired redox homeostasis are important factors in the frailty process [187]. Vitamin E is the main lipophilic antioxidant involved in these mechanisms [126]. Furthermore, Sakellariou et al. [188] showed a clear link between impaired redox homeostasis and age-related muscle atrophy and sarcopenia. Impaired skeletal muscle function and peripheral neuropathy or ataxia [53,131] (i.e., “ataxia with isolated vitamin E deficiency”) [189] have been reported in individuals with a low plasma concentration (below 8 or 12 µmol/L). A low vitamin E concentration was associated with a higher prevalence and higher risk of frailty [127]. Similarly, Rattray et al. [128] highlighted “four main metabolic areas that altered within the frailty ‘metabotype’ including the vitamin E pathway”. Mulligan et al. [60] found “significant positive associations between greater intakes of dietary vitamin E and indices of skeletal muscle mass, bone density status and total and hip fracture risk in both middle-aged and elderly men and women.” More generally, data from the FRAILOMIC initiative [129] revealed similar associations between frailty and low concentrations of α- and γ-tocopherol. These findings were in line with the results of the InCHIANTI cohort studies, in which low intakes of vitamin E were associated with frailty, but not to protein and energy intake [32,130]. The same researchers hypothesized that when few ROS are produced, only a small proportion of the α-tocopherol reacts to give α-tocopheroxyl radicals, and that greater ROS production involves a larger proportion of the α-tocopherol. In such a case, only a small proportion of the α-tocopherol is detected. Thus, vitamin E levels could be considered as “an indirect inverse marker of oxidative stress” [130] (Table 2).

Nevertheless, the results of interventional studies are somewhat incongruent. Fingeret et al. [123] showed that vitamin E and C supplementation neither improved grip strength nor prevented low-grip strength over a 5-year period in community-dwelling adults aged 40–80. However, Sachek et al. [132] found that vitamin E supplementation reduces exercise-induced oxidative stress and reduces muscle damage. Another study found that combined supplementation with vitamin E, vitamin D, and whey protein improved muscle mass and muscle strength in sarcopenic older adults [133].

In summary, a robust body of scientific evidence links vitamin E to frailty and sarcopenia. However, interventional trials have produced conflicting results. The true impact of supplementation requires further investigation.

## 5. Vitamin B12

Water-soluble vitamin B12 (cobalamin) is obtained exclusively from animal products in the diet [66]. Vitamin B12 uptake is an active, multistep process involving low gastric pH, gastric and pancreatic enzymes, gastric intrinsic factor (IF), and haptocorrin binders that bring the vitamin B12–IF complex to the cubulin receptor in the distal ileum. Upon internalization, a proportion of the unbound B12 vitamin is released into the plasma, where it binds to transport proteins [190]. Twenty to 30% of vitamin B12 plasma is bound to holotranscobalamin, the only form involved in cellular uptake. The other 70% to 80% is bound to haptocorrin, which is stored in the liver [191].

Depending on the diet (mostly animal products), the daily intake of vitamin B12 ranges from 5 to 7 micrograms a day, and the RDA for adults is often between 2 and 4 micrograms a day [66,68,69]. The RDA for older adults is 4 micrograms a day [66,69,70]. However, it has not been clearly established whether the dietary requirement for vitamin B12 changes with age [192].

Vitamin B12 marginal status and deficiency are currently defined as a serum cobalamin level between 148 and 221 pmol/L and below 148 pmol/L, respectively [66,67]. Furthermore, the serum vitamin B12 concentration may not reflect the functional vitamin B12 status [138]. Vitamin B12 deficiency leads to the accumulation of homocysteine and methylmalonic acid (MMA), which might be better diagnostic biomarkers [138]. In people with normal renal function, an elevation of MMA (210–450 nmol/L) [66] is often considered to be the most specific biomarker of vitamin B12 deficiency. Wolffenbuttel (2020) [144] showed that high serum MMA concentration was associated with poor functional performance, whereas an elevated serum MMA concentration was not systematically associated with a low serum vitamin B12 concentration. The same researchers also found that a large proportion of individuals had a low serum B12 concentration and a normal MMA concentration [144]. Elevated homocysteine (>15 μmol/L) is a sensitive marker of vitamin B12 deficiency, but it is less specific since rises are also observed in cases of folate deficiency, vitamin B6 deficiency, and hypothyroidism [138,193].

The literature data suggest that the risk of vitamin B12 deficiency increases with age. In older patients, vitamin B12 deficiency is related to food-cobalamin malabsorption (70%) and loss of gastric intrinsic factor (IF). Low dietary intake is less frequent, although vegetarian or vegan diets tend to increase this risk [71]. Food-cobalamin malabsorption is primarily caused by gastric atrophy (whether related or not to a Helicobacter pylori infection) but can also be due to the consumption of certain medications [194], such as the widely prescribed proton pump inhibitors, H2-receptor antagonists, and metformin [195]. In the Framingham study, the prevalence of vitamin B12 deficiency among community-dwelling older adults was 12% [74]. Other studies of institutionalized older people have reported higher values between 30% and 40% [196]. More recently, the prevalence of vitamin B12 deficiency in the UK and the USA was estimated to be around 6% in people under 60 and close to 20% in those over 60 [73]. The lack of “gold standard” tests and cut-offs can be explained by the uncertainty regarding (i) the consequences of vitamin B12 deficiency and (ii) the effectiveness of supplementation [197] (Table 1).

The diagnosis of vitamin B12 deficiency is difficult for several reasons. First, the symptoms (paresthesia, muscle cramps, ataxia, asthenia, and cognitive and psychiatric disorders) are often non-specific [149,150]. Second, the prevalence of megaloblastic anemia is lower than anticipated [196]. Vitamin B12 has an important role in the synthesis of myelin [198]. Thus, vitamin B12 deficiency may cause peripheral neuropathy (subacute combined degeneration of the spinal cord) [149,150]. These demyelinating disorders commonly lead to peripheral neuropathy with muscle weakness and pain in the distal limbs. All these neurological symptoms lead to balance disorders and (in some cases) gait ataxia, which can lead to falls and loss of mobility [151].

A low serum vitamin B12 level has an effect on physical performance, gait speed, and balance [142]. O’Leary et al. [199] suggested that vitamin B12 deficiency is associated with the length of hospital stay. However, this association is subject to debate. In the Korean Frailty and Aging Cohort Study of 2938 older people, vitamin B12 deficiency did not directly increase the incidence of frailty [141]. Nevertheless, MMA concentrations were independently associated with poor functional status, physical performance, and muscle strength, and these associations were stronger than for the serum vitamin B12 concentration [144].

Vitamin B12 and the other B vitamins are involved in the response against oxidative stress [134]. In a recent systematic review, van de Lagemaat et al. [135] suggested that lower vitamin B12 status is related to elevated pro-oxidant levels and low antioxidant levels; these factors have been integrated into models of cellular aging and are correlated with age-related diseases. Regarding the most common biomarkers of oxidative stress (glutathione, malondialdehyde, total antioxidant capacity, and total oxidant status), most studies included in this systematic review reported significantly higher oxidative stress when vitamin B12 status was low. These results should be interpreted with caution and require further studies [135].

A key point is that vitamins B12, B6, and B9 help to reduce serum levels of homocysteine. Vitamin B12, B6, or B9 deficiency impairs the remethylation of homocysteine, causing an elevation of the plasma homocysteine level [136]. Correcting the intake of vitamin B6/B9/B12 reduces the plasma homocysteine level [153]. Ford et al. [154] observed that an increase in plasma vitamin B6 and B12 levels led to a reduction in plasma homocysteine levels. The researchers concluded that high-dose supplementation with B multivitamins might promote the breakdown of homocysteine to a greater extent than folate. Through its involvement in homocysteine metabolism, vitamin B12 has an essential role in DNA methylation. Vitamin B12 is a cofactor for methionine synthase, the enzyme that catalyzes the formation of methionine from homocysteine [137]. Indeed, Yadav et al. [155] observed that vitamin B12 supplementation increased overall DNA methylation and influenced the regulation of several genes. An elevated serum homocysteine level is associated with an increased risk of decline in physical function [145] and disability in ADL and instrumental ADL [146,147]. The association between homocysteine and disability may be explained by the compound’s effects on quadriceps strength and usual gait speed [146]. In a nutritional epidemiology study of older population cohorts, Behrouzi et al. [143] showed that vitamin B group intake was directly and positively associated with physical functioning, as assessed by the total Short-Physical Performance Battery score and the chair stand test score. Another study showed that homocysteine concentrations are inversely associated with physical performance (e.g., gait speed) in older adults [148]. Moreover, hospitalized older patients with elevated plasma homocysteine levels may have an increased risk of decline in physical function [147]. Marengoni et al. [147] concluded that (i) the prevalence of hyperhomocysteinemia in hospitalized patients is very high, even in patients with normal cobalamin and folate concentrations, and (ii) vitamin supplements significantly reduce serum homocysteine concentrations, even when serum vitamin concentrations are normal [147,200]. Furthermore, Grootswagers et al. [139] suggested that an adequate intake of B vitamins (B6, B9, and B12) can improve mitochondrial function, which may be beneficial for physical performance. Indeed, B vitamins are involved in mitochondrial metabolism [201], which can be impaired by elevated homocysteine levels [202]. Lastly, Swart et al. [203] suggested that homocysteine is associated with elevated mortality and nursing home admission risk in women. However, low serum vitamin B12 levels appear to be correlated with an increased prevalence of Alzheimer disease [152], and high serum levels of MMA and homocysteine are related to brain atrophy, white matter lesions [197], and lacunar stroke [204]. However, lowering the homocysteine plasma concentration via vitamin B supplementation does not appear to improve cognitive functions [205]. All these roles of vitamin B12 are subject to intense research, and further investigation of some of the controversial results is essential (Table 2).

Due to vitamin B12′s many roles in cellular metabolism and clinical impairments, this topic is likely to remain a major area of interest in aging research and clinical practice in older populations.

## 6. Vitamin B9

Vitamin B9 is a generic term for a family of water-soluble compounds whose common activity is based on the parent structure of folic acid [156]. Humans cannot synthesize folates and depend on dietary intake. Folate is the naturally occurring form of vitamin B9. Unlike vitamin B12, folate reserves are low and have to be maintained by daily intake. Folate is found mainly as polyglutamate, a form that is naturally present in green vegetables, peanuts, and legumes, notably. The RDA ranges from 200 µg/day to 400 µg/day for adults and 330 to 400 µg/day for older adults [77]. According to the National Institutes of Health, the RDA for adults is 400 µg of dietary folate equivalents (a unit that accounts for the greater bioavailability of folic acid) [76]. According to the EFSA (2014), erythrocyte folate concentrations below 340 nmol/L and serum folate concentrations below 10 nmol/L indicate folate deficiency [77]. In a study of community-dwelling older adults in Western countries, Borg et al. [206] reported that 29% of the men and 35% of the women had an inadequate folate intake. However, a recent review indicated that “the mean values reported for erythrocyte folate and serum folate in older adults across Europe are above the cut-offs proposed by the EFSA”, and the reviewers did not find evidence of folate deficiency [207]. Among older people, the main causes of deficiency are inadequate dietary intake, undernutrition, alcoholism, malabsorption (intestinal diseases), and drug interactions [72]. Due to a low dietary intake, 9–12% of older people in the UK suffer from vitamin B9 deficiency (Table 1).

Folate has a key role in many metabolic and cellular pathways. Some of these pathways are critical, including DNA/RNA/protein methylation, DNA replication, repair, and pathways involving the synthesis of nucleotides, amino acids, and some vitamins [156,157]. A meta-analysis suggests that folate supplementation leads to less oxidative stress by increasing serum glutathione (GSH) concentrations and total antioxidant capacity and decreasing serum concentrations of malondialdehyde (MDA) [159]. Folate has an important role in cellular functions (especially DNA methylation), and it has been suggested that these pathways are associated with carcinogenesis [157]. Vitamin B9 deficiency causes several disorders (including megaloblastic anemia, neuropathy, and neural tube defects) [162] but is also involved in chronic diseases, such as Alzheimer’s disease [163,164], cardiovascular disease [165], depression [166], and muscle weakness, in older people [161]. Bartali et al. suggested that low intake of vitamin B9 was associated with frailty [32].

Lastly, vitamin B9 is (like vitamin B12) involved in the metabolism of homocysteine. Vitamin B9 deficiency leads to elevation of the serum homocysteine concentration [158]. Thus, the intakes of vitamin B9 and B12 also appear to be associated with physical functioning (see the section above on vitamin B12) (Table 2).

## 7. Vitamin B6

Pyridoxal 5′-phosphate is the active form of the water-soluble vitamin B6, and levels of this compound are regulated by the liver. Vitamin B6 is not synthetized by humans and is provided by different foods, such as meat, milk products, potatoes, nuts, beans, and several fruits and vegetables [208]. According to the EFSA (2016), plasma pyridoxal-phosphate (PLP) concentration below 30 nmol/L indicates vitamin B6 deficiency [79]. For vitamin B6, the EFSA (2016) [79] recommends population reference intakes of ≥1.7 mg/day for men and ≥1.6 mg/day for women. Among older people, the RDA rise to 2.2 mg/day [79,80]. The main causes of deficiency are increased requirements in aging, inadequate dietary intake, undernutrition, alcoholism, and pyridoxine-inactivating drugs, liver disease, renal dialysis, rheumatoid arthritis, and HIV [72]. A systematic review of studies of community-dwelling older adults in Western countries [206] reported that 31% of the men and 24% of the women had an inadequate B6 intake.

In Michelon et al.’s study of older women, the overall prevalence of vitamin B6 deficiency was 41.6% [84]. Although the overall prevalence of vitamin B6 deficiency in the older population is not well established, some epidemiological studies have found a high value (from 16% to 48% in community-dwelling older people and up to 75% in institutionalized older people) [79,82,85,86,87] (Table 1).

Vitamin B6 is involved in many metabolic and physiologic pathways. Like vitamins C and E, vitamin B6 is a powerful antioxidant [168]. Vitamin B6′s ability to neutralize ROS makes it an important micronutrient in the aging process. In a recent study [169], researchers found that pyridoxine (vitamin B6) can promote GSH biosynthesis via the PKM2-Nrf2 pathway. This function participates in the important cellular antioxidant role of pyridoxine via GSH biosynthesis. Michelon et al.’s study of non-frail, prefrail, and frail older women found that frailty was associated with low levels of vitamins B6, E, and D and carotenoids [84].

Vitamin B6 is a key component of the immune system and is therefore essential for normal immune reactions [170]. The vitamin is also involved in cytokine metabolism and antibody production. Decreases in lymphocyte proliferation and antibody and IL-2 production are observed in patients with vitamin B6 deficiency [167] (Table 2).

Lastly, vitamin B6 is involved in the metabolism of homocysteine. Hence, vitamin B6 deficiency causes an increase in the serum homocysteine concentration. The intake of vitamin B6 (like vitamins B12 and B9) may also be associated with physical functioning (see the section above on vitamin B12).

## 8. Discussion

Undernutrition and macronutrient deficiencies are widely reported as having a negative impact on functional recovery in older adults. However, vitamins have major roles in the health of older adults. The vitamins’ involvement in oxidative, inflammatory, immune, genetic and epigenetic regulation mechanisms make them essential for cellular homeostasis.

Molecular aging has been defined in terms of nine hallmark features: an accumulation of DNA damage, telomere shortening, epigenetic alterations, loss of proteostasis, deregulated nutrient sensing, mitochondrial dysfunction (responsible for oxidative stress), cellular senescence, stem cell exhaustion, and altered intercellular communication [209]. All these molecular processes sustain chronic, low-grade inflammation that impairs tissue repair and regeneration, which in turn leads to the release of inflammatory cytokines and acute-phase reactants into the circulation [210]. During aging, this chronic inflammation contributes to the pathogenesis of age-related diseases (including sarcopenia) and chronic diseases that can accentuate the inflammatory aging process [24]. This interplay contributes to the loss of physical function, inability to perform ADL, and the frailty observed in older subjects.

Above, we noted that both fat-soluble vitamins (vitamins D and E) and water-soluble vitamins (vitamins B6 and C) have fundamental redox roles. We have also reviewed the robust body of literature data on the involvement of specific micronutrients in immune and inflammatory functions. Other vitamins not detailed in this review are involved in related-age disabilities and participate in the pathogenesis of frailty and sarcopenia. Similar to vitamins C and E, vitamin A and its precursor carotenoids are dietary antioxidant vitamins. In a cross-sectional study, an association was shown between higher plasma carotenoids levels and higher strength measures [211]. Semba et al. [212] confirmed in a longitudinal study that plasma carotenoids level was associated with sarcopenia and were an independent predictor of severe walking disability progression in the population of older women living in the community. These interesting results are not clinically significant in older people from high-income countries because vitamin A deficiency is not an issue in this population. All these aspects of cellular metabolism emphasize the importance of vitamins in the aging process. However, clinical trials on supplementation with one or more of these vitamins (either alone or combined as multivitamins and minerals) have been mainly given disappointing results for functional outcomes.

Periods of immobilization and reduced physical activity may accompany acute pathologies, trauma, hospital-associated deconditioning (secondary sarcopenia), and aging (primary sarcopenia). Nutrition can have a beneficial impact on physical activity through the provision of key nutrients with antioxidant properties (i.e., affecting molecular pathways in the skeletal muscles and epigenetic regulation). Thus, nutrition is not simply a source of energy [213]. Episodes of immobilization and reduced physical activity lead to a loss of muscle mass, which might worsen the older patient’s underlying disease or increase the level of frailty [214,215]. Skeletal muscle is the main protein source mobilized by the body when faced with organic stress. Thus, the muscle atrophy related to immobilization is accentuated by an underlying sarcopenic state and becomes harmful for older patients undergoing rehabilitation.

The optimal strategy in older patients cannot be a “one-size-fits-all” approach. Nutritional programs enhance the effects of muscle strength interventions and thus maximize functional recovery. Furthermore, inflammaging has been incorporated in models of aging [216,217] and physical decline [218]. Thus, there are two schools of thought in this field. The anti-inflammatory effect (even if indirect) of particular micronutrients might combine with that of physical exercise [219,220]. The improvements in muscle strength and physical capacities and the correction of nutritional deficiencies might work together synergistically in the prevention of frailty [221]. Nevertheless, it is important to bear the limitations of these approaches in mind. Although exercise is a key link in the gain/maintenance of muscle reserves, performance may be compromised in older adults. Isolated nutritional care for rehabilitation management (with a combination of macro- and micronutrients) can optimize functional recovery [222].

Along with the specific interventions in older adults described above, the other crucial issue is the early diagnosis of undernutrition. As exposed above, vitamin deficiencies are associated with frailty and have a deleterious role in the pathogenicity of sarcopenia. Older hospitalized patients present a high prevalence of frailty and malnutrition. Then it seems appropriate to detect vitamin deficiencies in these conditions. However, the effects of supplementation are often questioned, and further investigations are required. As the first step in the nutrition care process, malnutrition screening is useful for identifying older people who are malnourished or at risk of malnutrition. The early identification of malnutrition can improve physical function and reduce the length of hospital stay [223]. In some countries, the frequency of malnutrition screening among older (>75) people depends on the type of residential setting: annual screening by the family physician for community-dwelling adults and weekly screening in hospitals [4]. Nutrition is also a component of an individual’s intrinsic capacity, as explored by the World Health Organization’s Integrated Care for Older People (ICOPE) program [224]. The first step in the ICOPE care pathway is a screen for declines in intrinsic capacity among community-dwelling older adults, with the questions “Have you unintentionally lost more than 3 kg over the last three months?” and “Have you experienced a loss of appetite?”. The second step is to undertake a person-centered assessment of nutritional status in primary care, using the Mini Nutritional Assessment. Depending on the results, a personalized care plan can be developed [225]. Undernutrition is associated with micronutrient deficiencies. Improving undernutrition screening and diagnosis might also improve the detection and correction of micronutrient deficiencies. However, the implementation of policies of prevention as well as improved screening strategies to ensure adequate dietary intake in the older population is a priority. For example, the European Society for Clinical and Economic Aspects of Osteoporosis, Osteoarthritis, and Musculoskeletal Diseases (ESCEO) working group (2016) recommends, in addition to routine screening and early diagnosis, preventive actions to promote dietary quality and a physically active lifestyle [22].

## 9. Conclusions

Although vitamin deficiencies (whether combined or not with undernutrition) have a serious impact on healthy aging in older people, physicians are not sufficiently aware of this (potentially reversible) issue. Further comprehensive research programs on nutritional interventions are needed, with a view to reducing deficiencies in older people and thus decreasing the risk of frailty and sarcopenia. We still need a better understanding of (i) the role of each vitamin in the aging process, (ii) the interaction between vitamins, and (iii) how vitamin deficiency contributes to the onset of frailty.

A multimodal intervention might be an interesting approach to the fight against age-related diseases and frailty. Based on our fundamental knowledge of the metabolic pathways involving vitamins, a focused nutritional approach might enable better diagnosis of nutritional deficiencies and the initiation of appropriate clinical interventions (rehabilitation, physical activity, cognitive activities, etc.) for reducing the risk of frailty. Even though synergistic supplementation and multimodal approaches appeared to be beneficial, further interventional studies are required—despite the costs and difficulties associated with this type of research.

## Figures and Tables

**Table 1:** Sources, dietary allowances, and deficiencies in selected vitamins.

Vitamin	Main Sources	Ranges and Concentrations for Deficiency	RDA for Adults	RDA for Older Adults	Main Causes of Deficiency in Older Adults	Prevalence of Deficiency or Insufficiency in Adult Populations	Prevalence of Deficiency or Insufficiency in Older Populations
Vitamin D	Fatty fish, oily fish, fish liver, red meat, liver, egg yolks, cheese, milk, mushrooms; Sun exposure [36,37]	Deficiency < 30 nmol/L *, Inadequate below 30–50 nmol/L *, Sufficient > 50 nmol/L * [39]; Insufficiency 20–29 ng/mL *, Deficiency < 20 ng/mL (50 nmol/L) * [34];	200–800 IU/day (5–20 µg) [37,39]	400–800 IU/day (10–20 µg) [37,39]	Lack of sun exposure/Poor skin integrity/Fat malabsorption or maldigestion/Low dietary intake/Reduced renal function/Medications [40]	8% to 17% of women and 14.2% to 15.6% of men (<25/30 nmol/L) [41,42]; 28.2% to 57.8% of women and 39% of men (<45/50 nmol/L) [41]	47% to 56% of women and 36% to 45% of men [43,44]; 17% < 25 nmol/L and 67% < 50 nmol/L (mean age 71.8 years) [45]; 90% of older patients in rehabilitation centers [46,47]; 90% of institutionalized women (<50 nmol/L) compared with 57% of non-institutionalized women [48]; 80% in nursing homes (<50 nmol/L) [49]
Vitamin C	Fruits, vegetables, potatoes, animal kidney and liver [50]	Deficiency < 11.4 μmol/L [51]; adequate > 28 μmol/L, suboptimal 11–28 μmol/L, deficient < 11 μmol/L [52].	40–110 mg/day [50,53,54]	75–120 mg/day [50,53]	Low/inadequate dietary intake, undernutrition, malabsorption (intestinal diseases), inflammation, smoking, alcohol abuse, drug abuse [55]	1.0% to 20% of adults (high-income countries) [56,57]	9–14% of older people (high-income countries) [56,58,59]
Vitamin E	Vegetable oils, nuts, seeds, wheat germ and some vegetables like avocado and spinach [60,61,62]	Deficiency < 12 μmol/L (α-tocopherol) (EFSA) [63];	15 mg (35 µmol)/day [53];	15 mg (35 µmol)/day [53];	Fat malabsorption, fat metabolism disorders, mutations in the tocopherol transfer protein, and isolated vitamin E deficiency syndrome (rare) [64]	0.1% of people (USA) [64]; 0.7% to 55% of people (developing countries) [65]	N/R
Vitamin B12	Mainly from animal products in the diet [66]	Serum cobalamin < 148 pmol/L and marginal status 148–221 pmol/L (serum cobalamin); Serum holotranscobalamin < 21–45 pmol/L; Serum total homocysteine > 15 μmol/L (hyperhomocysteinaemia); Serum methylmalonic acid > 210–450 nmol/L [66,67]	2–4 µg/day [66,68,69];	4 µg/day [66,69,70]	Cobalamin malabsorption (intestinal diseases, gastric/intestinal resection, atrophic gastritis, pancreatic insufficiency) (70%) and loss of gastric intrinsic factor (dietary intake deficits are less frequent), alcoholism, drug interactions [71,72]	3 to 6% of people aged under 60 [67,73];	6% to 20% of people aged 60 to 70 [67,73,74]; 30% to 40% of institutionalized older people [75].
Vitamin B9	Vegetables (asparagus, brussels sprouts, spinach, etc.), fruits, rice, nuts, beef liver, etc. [76]	Deficiency < 340 nmol/L for erythrocyte folate and <10 nmol/L for serum folate [77]	200–400 µg/day for women, 200–400 µg/day for men [77]	330–400 µg/day [77]	Low/inadequate dietary intake, undernutrition, alcoholism, malabsorption (intestinal diseases), drug interactions [72]	Approximately 20%, and ranges from 13.5% to 26.6% in woman (USA) [78]	9–12% (UK) [72]
Vitamin B6	Grains, pulses, nuts, seeds, potatoes, some herbs and spices, meat and meat products [79]	Deficiency < 30 nmol/L (serum PLP) [79]	1.2–1.5 mg/day for women and1.3–1.8 mg/day for men [79,80,81]	1.2–2.2 mg/day for women and 1.4–2.2 mg/day for men [79,80]	Low/inadequate dietary intake, undernutrition, alcoholism, pyridoxine-inactivating drugs, liver disease, dialysis, rheumatoid arthritis, HIV, increased requirements with age [72]	0.5% to 5% of adults [79,82,83]	41.6% of older woman [84]; 16% to 48% of community-dwelling older people [79,82,85,86,87]; 75% of institutionalized older people [85]

* Serum 25-hydroxy vitamin D (25-OHD) levels PLP = pyridoxal-phosphate. RDA: Recommended dietary allowance.

**Table 2:** Functions, pathways, and consequences of deficiencies for selected vitamins, MMA = Methylmalonic acid, ADL = Activities of daily living.

Vitamin	Main Cellular Metabolic Pathways	Functions	Associated Geriatric Syndromes and Diseases			Effect of Supplementation *
			Frailty	Sarcopenia/Functional Performances and Deficiency	Other Syndromes and Diseases	
**Vitamin D**	Gene regulation (cellular metabolism, regulation of innate and adaptive immunity) [98]; Immunomodulatory properties [99]; Direct regulatory role in skeletal muscle [93];	Maintains calcium and phosphorus homeostasis in the circulation, cell differentiation, and antiproliferative actions in bone marrow (osteoclast precursors and lymphocytes), immune system cells, skin, breast and prostate epithelial cells, muscle and intestine [37]; Oxidative stress (antioxidant agent) [95]	Increased risk of frailty [91]; Associated with the severity of frailty [92]	Association with low muscle mass and function, poor physical performance, risk of falls [88,89], lower grip strength 3 years later [90], decrease in walking time or time to complete a repeated chair stand [88]	Associated with adverse clinical outcomes during the hospital stay and after 30 days (mortality, length of stay, falls, and quality of life) [100]; Increased risks of incident hospital-diagnosed delirium [101]; Cardiovascular disease [102]; Association with poorer cognition [103]	Rehabilitation outcomes after acute stroke not improved [104]; Data are incongruent, and further evidence of clinical benefit is required [34,105,106]; Levels ranging approximately from 75 to 120 nmol/L would increase in rates of all-cause mortality, some cancers, risk of cardiovascular events, falls and fractures among older adults [36,39]; High-dose administration of vitamin D3 to deficient older women increased total antioxidant capacity levels [97]
**Vitamin C**	Genetic and epigenetic regulation mechanisms (hydroxylation of transcription factors, tRNA and ribosomal proteins, demethylation of DNA, and histones), oxidative stress (protection against ROS), reduction in iron in the gastrointestinal tract, inflammation (pro-inflammatory cytokines production) [55,107,108,109]	Reducing and antioxidant agent;Biosynthesis of collagen, carnitine, and catecholamine; metabolism of cholesterol, steroids, hormones (oxytocin, vasopressin, cholecystokinin, and alpha-melanocyte-stimulating hormone), hydroxylation of phenylalanine in tyrosine formation) [50]	Association of lower concentrations of antioxidants (e.g., vitamins C and E) with frailty [110]; Association of a lower intake with a higher risk of frailty [111]	Positive association between circulating vitamin C levels and measures of skeletal muscle mass [112]	Scurvy [113]; Association of high vitamin C status with better cognitive function [114,115,116,117,118]; Association of vitamin C status with all-cause mortality and association of vitamin C status with cancer and cardiovascular disease mortality [119,120,121]	Effective against scurvy [107,113]; No difference in cognitive function between supplementation and adequate dietary intake [118]; No reduction or only a moderate reduction in the risk of heart disease [122]; Neither an improvement in grip strength nor prevention of low-grip strength [123]; Potentially beneficial for increasing DNA repair [124]
**Vitamin E**	Modulating gene expression in the brain, transduction and gene expression, survival factor for specific neuronal cells, antioxidant preventing damage to membranes or proteins and regulating their activity by specifically scavenging reactive oxygen and nitrogen species [125]	Lipophilic antioxidant, intracellular and cellular membrane integrity and stability, stability of erythrocytes, conductivity in central and peripheral nerves, and prevents hemolytic anemia [63,126]	Association of low vitamin E concentration with a higher prevalence and higher risk of frailty [127,128,129,130];	Positive associations between greater intakes and indices of skeletal muscle mass, bone density status, and the total and hip fracture risks [60]	Peripheral neuropathy or ataxia [53,131]	Neither an improvement in grip strength nor prevention of low-grip strength [123]; Reduction in exercise-induced oxidative stress and muscle damage [132]; Combined supplementation with vitamin E, vitamin D, and whey protein improved muscle mass and muscle strength [133]
**Vitamin B12**	Antioxidative response against oxidative stress [134,135]; Metabolism of homocysteine [136] and a cofactor for methionine synthase [137]; Metabolism of MMA [138]; Mitochondrial function [139]	Cell division, red blood cell formation, energy metabolism, immune system function, neurological and psychological functions, reduction in tiredness and fatigue [136,140]	Does not directly increase the incidence of frailty [141]	Physical performance; gait speed and balance [142]; Directly and positively associated with physical functioning [143]; Association between MMA concentrations and functional status, physical performance and muscle strength [144]; Elevated serum homocysteine level is associated with an increased risk of decline in physical function [145] and with disability in ADL and instrumental ADL [146,147]; Homocysteine concentrations are inversely associated with physical performance (e.g., gait speed) in older adults [148]	Peripheral neuropathy, paresthesia, muscle cramps, ataxia, asthenia, cognitive and psychiatric disorders [149,150]; Ataxia, which can lead to loss of mobility and falls [151]; Alzheimer’s disease [152]; Megaloblastic anemia [72]	Reduction in plasma homocysteine levels [153,154]; Increases overall DNA methylation and influences the regulation of several genes [155]; Adequate intake of B vitamins (B6, B9 and B12) can improve mitochondrial function, which may be beneficial for physical performance [139]
**Vitamin B9**	DNA, RNA, and protein methylation (DNA replication, repair, and pathways involving the synthesis of nucleotides, amino acids, and some vitamins) [156,157]; Metabolism of homocysteine [158]; Oxidative stress (antioxidant agent) [159]; Mitochondrial function [139]	Cell division and tissue growth, red blood formation, psychological functions, reduction in tiredness and fatigue; amino acid synthesis, immune system [77,158,160]	Association between low intake and frailty [32]	Muscle weakness in older people [161]; Directly and positively associated with physical functioning [143]	Megaloblastic anemia [72], neuropathy and neural tube defects [162]; Alzheimer’s disease [163,164]; Cardiovascular disease [165]; Depression [166]	Reduction in plasma homocysteine levels [153,154]; Less oxidative stress by increasing serum GSH concentrations and TAC, and decreasing serum concentrations of MDA [159]
**Vitamin B6**	Cofactors for enzymes involved in amino acid metabolism, one-carbon reactions, glycogenolysis and gluconeogenesis, haem synthesis, niacin formation, lipid metabolism, neurotransmitter synthesis, and hormone action [79]; Metabolism of homocysteine [153,154]; Cytokine metabolism, antibody production, and lymphocyte proliferation [167]; Antioxidant agent [168] cellular antioxidant role of pyridoxine via GSH biosynthesis [169]; Mitochondrial function [139]	Protein and glycogen metabolism, nervous system function, red blood cell formation, immune system function, regulation of hormonal activity [170]	Association between increased levels of vitamin B6 and frailty [84]	Directly and positively associated with physical functioning [143]	Microcytic anemia, confusion, depression, inflammation of the tongue and sores or ulcers of the mouth, dermatitis [72]	Reduction in plasma homocysteine levels [153,154]

* No consensus on supplementation for people with deficiencies except for vitamin D.
